# Extrauterine adenomyoma of the lesser omentum: A case report and review of the literature

**DOI:** 10.1097/MD.0000000000030240

**Published:** 2022-09-09

**Authors:** Yanlin Chen, Liangyong Deng, Jingbo Zhao, Tianwen Luo, Zhong Zuo

**Affiliations:** a Department of Pathology, Women and Children’s Hospital of Chongqing Medical University, Chongqing, China; b Department of Pathology, Jinshan Hospital, The First Affiliated Hospital of Chongqing Medical University, Chongqing, 401122, China; c Anbiping (Chongqing)Pathological Diagnosis Center, Chongqing, China.

**Keywords:** abdominal surgery, extrauterine adenomyoma, gastric carcinoma, lesser omentum, lymph node-like mass

## Abstract

The extrauterine adenomyoma is rare and it is extremely rare outside the pelvic cavity. Herein, we reported the first case of a single extrauterine adenomyoma occurring in the lesser omentum.

**Patients concerns:**

This case involved a 55-year-old woman who had undergone subtotal gastrectomy and omentectomy for gastric carcinoma. During postoperational pathological examination, 1 lymph node-like mass was coincidentally found in the lesser omentum. The patient had a history of hysterectomy for uterine leiomyoma 8 years ago.

**Diagnoses:**

The resected 17 “lymph nodes” from the lesser omentum were routinely checked for possible metastasis of gastric carcinoma. One of lymph node-like mass was microscopically showed that it was composed of benign smooth muscle components, endometrial glands and stroma by HE staining. Therefore, adenomyoma was initially considered.

**Interventions:**

The lymph node-like mass was removed together with the lesser omentum during the subtotal gastrectomy and omentectomy for gastric carcinoma. No special intervention was performed for the adenomyoma.

**Outcomes:**

Immunohistochemical staining confirmed that smooth muscle tissue was diffusely and strongly positive for Desmin, smooth muscle actin, estrogen receptor, and progesterone receptor, and negative for CD117, Dog-1, S100, and CD34. Endometrial glands and stroma were positive for estrogen receptor and progesterone receptor, and the endometrium interstitium was also positive for CD10. The final diagnosis of extrauterine adenomyoma occurring in the lesser omentum was established.

**Lessons:**

So far, to the best of our knowledge, total 53 cases of extrauterine adenomyoma have been reported in 45 English reports. The most common location for a single mass was pelvic cavity (37 cases), but rarely outside the pelvic cavity. This is the first case of a single extrauterine adenomyoma occurring in the lesser omentum.

## 1. Introduction

It is well known that the presence and growth of functioning endometrial tissue in places other than the endometrium are called endometriosis. Adenomyosis will be called when the endometrial tissue is within the myometrium of the uterus, and endometriosis will be called when ectopic endometrial tissue appears outside the uterus.^[1]^ Adenomyoma is a focal and localized mass with adenomyosis-like structure. It is rarely located outside the uterus, which is termed as extrauterine adenomyoma.^[1]^ Adenomyoma is mainly composed of smooth muscle tissue, endometrial glands, and stroma.^[1]^ The extrauterine adenomyoma is rare, and it was first reported by Rubenstein and Kurzon in 1952.^[2]^ So far, the reported sites of extrauterine adenomyoma are mainly in the pelvic cavity, such as pararectal space,^[1,3–6]^ ovary,^[1,7–15]^ broad ligament,^[16–23]^ the round ligament,^[1,2,24]^ paraovarian,^[10,25–27]^ parametrial,^[10]^ and pelvic wall.^[28]^ It is extremely rare outside the pelvic cavity such as liver,^[29,30]^ upper abdomen,^[6]^ inguinal region,^[31,32]^ colon,^[33,34]^ appendix,^[35]^ small bowel mesentery,^[36,37]^ and retroperitoneal space.^[38]^ Rare cases of extrauterine adenomyomas in multiple places were also reported.^[1,6,11,39–41]^ It is nearly impossible to confirm the diagnosis based on clinical manifestations and imaging examination alone, and the diagnosis can only be confirmed by pathological examination.

To the best of our knowledge, this is the first case of a single extrauterine adenomyoma occurring in the lesser omentum. It was coincidentally discovered in a woman who underwent subtotal resection of gastric cancer and resection of greater omentum and lesser omentum. The patient had a history of laparoscopic myomectomy. In this paper, we report this rare case and review literature to provide insight into the clinical features, diagnosis, and pathogenesis of this rare disease.

## 2. Case report

### 2.1. Clinical findings

A 55-year-old Chinese woman presented with abdominal cramps, nausea, and vomiting with no obvious causes. The vomit was black contents, and her stools were tarry. In an outpatient clinic of our hospital, upper gastrointestinal endoscopy revealed an ulcer with a size of 1.2 × 0.6 cm in the angle of the stomach. Biopsy from the ulcerated lesion revealed poorly differentiated adenocarcinoma. The patient was admitted to our hospital and the laboratory findings on admission were within normal limits. Based on these findings, the patient was scheduled for subtotal resection of gastric cancer and resection of greater and lesser omenta with contained lymph nodes.

The patient had undergone hysterectomy for uterine leiomyoma 8 years ago, and none of adenomyosis or adenoma inside or outside of uterus was found. Furthermore, there were no reports of abnormalities after the operation follow-up.

### 2.2. Pathology findings

**Figure 1.**
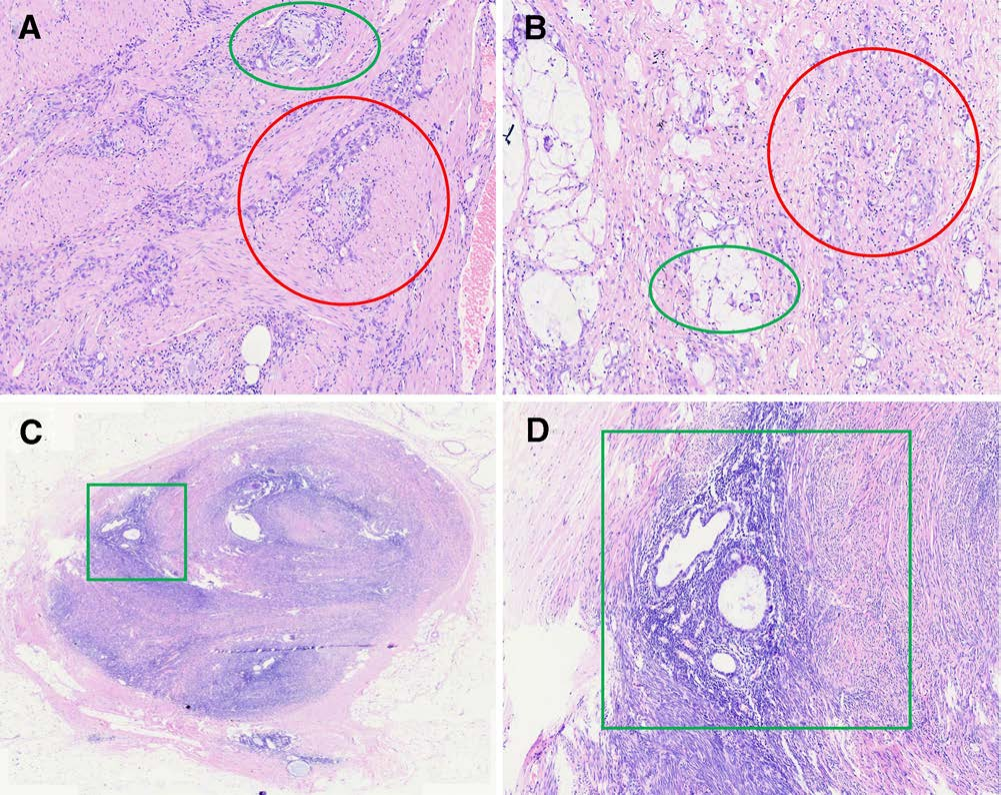
Histological examination by HE staining. Histological examination revealed poorly differentiated adenocarcinoma of the stomach (A), which invaded nerves (green circle) and muscle layers (red circle). As shown in (B), the cancer tissue consisted of poorly differentiated adenocarcinoma (red circle) and mucinous adenocarcinoma (green circle). (C, D) The histological features of lymph node-mass in the lesser omentum are shown. (D) A partial amplification of (C). The tumor was composed of benign smooth muscle tissue, endometrial glands, and stroma (D). HE = hematoxylin and eosin.

In the resected specimens, there was an ulcer of 1.1 × 0.5 cm in the angle of the stomach, supporting the preoperative diagnosis. The cut surface of the ulcer-located stomach wall was gray-white with slight hard texture. The lesion microscopically revealed poorly differentiated adenocarcinoma with invading muscle layer and nerve bundles (Fig. 1A, B) and some mucinous adenocarcinoma differentiation was evident (approximately 20%; Fig. 1B).

**Figure 2.**
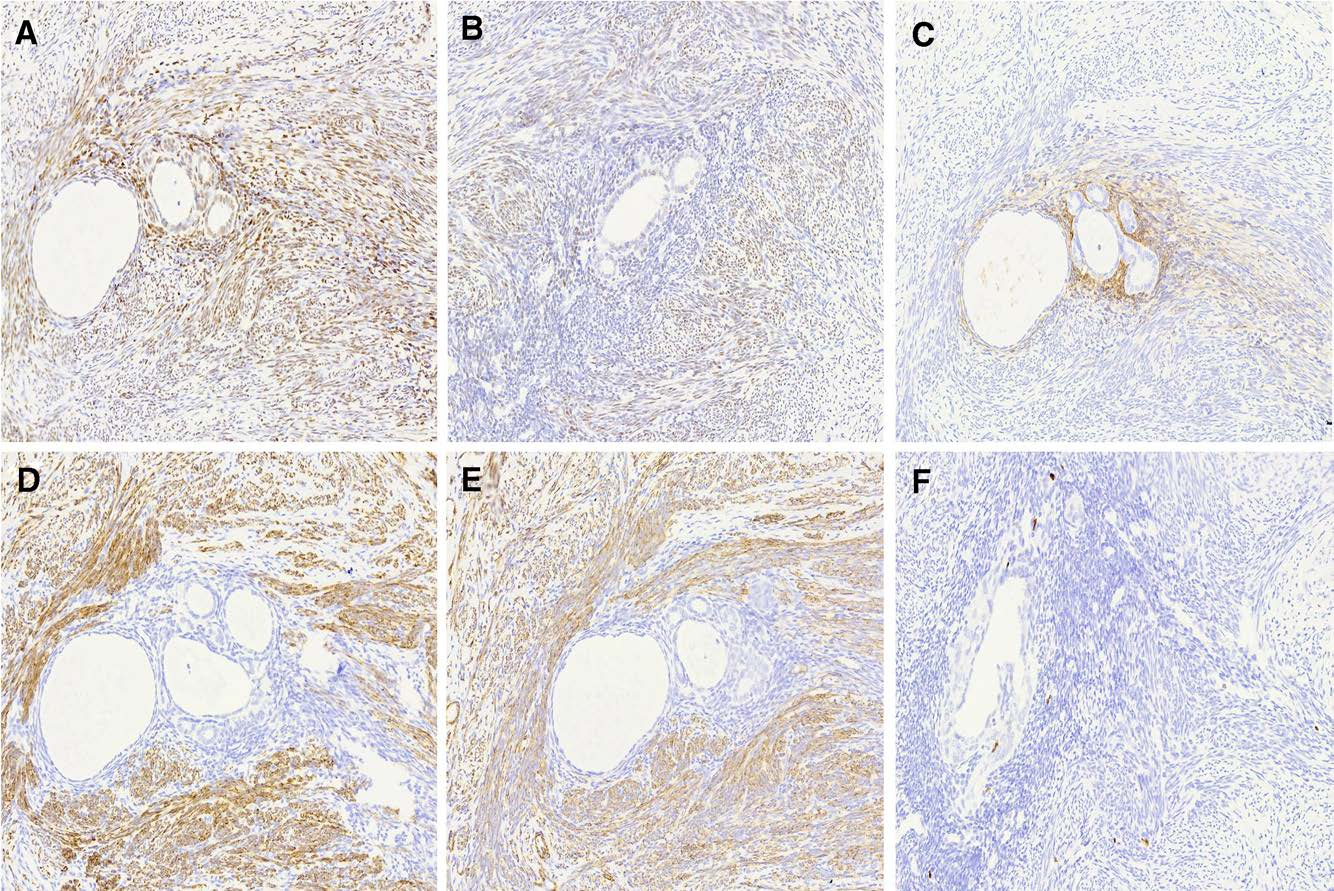
Immunohistochemical staining. Immunohistochemical staining for ER (A), PR (B), CD10 (C), Desmin (D), SMA (E), and Ki-67 (F) was performed. The smooth muscle tissue was positive for ER, PR, Desmin, and SMA and negative for CD10, while the endometrial glands and stroma were positive for ER and PR and negative for Desmin and SMA. The endometrial interstitium was positive for CD10. The expressions of Ki-67 in the smooth muscle tissue, endometrial glands, and interstitium were very low (only a few cells are positive, <1%). CD10 = common acute lymphoblastic leukemia antigen, ER = estrogen receptor, PR = progesterone receptor, SMA = smooth muscle actin.

In order to check the possible carcinoma metastases of lymph nodes, the lesser and greater omenta were carefully investigated. A total of 17 small nodules with a diameter of 0.2 to 0.7 cm were found in the lesser omentum, while 17 small nodules with a diameter of 0.2 to 0.4 cm were found in the greater omentum. All small nodules in the greater omentum have been confirmed to be lymph nodes and showed no cancer metastasis (0/17), while 16 of 17 small nodules in the lesser omentum have been confirmed to be lymph nodes and one of them showed cancer metastasis (1/16). It is interesting to notice 1 small nodule with a size of 0.7 × 0.5 cm in the lesser omentum. It was grossly concerned as lymph node with cancer metastasis; however, it was microscopically shown that it is composed of smooth muscle tissue, endometrial glands, and stroma (Fig. 2C, D), where no atypia, significant mitotic activity, and necrosis of the smooth cells, glands, or stroma have been shown. From these histological features, the first impression diagnosis is adenomyoma rather than lymph node cancer metastasis.

In order to confirm the diagnosis, a series of immunohistochemical staining was performed including estrogen receptor (ER), progesterone receptor (PR), CD10, Desmin, smooth muscle actin (SMA), and Ki-67, CD117, Dog-1, CD34, and S100. The smooth muscle was positive for ER (Fig. 2A), PR (Fig. 2B), Desmin (Fig. 2D), and SMA (Fig. 2E), and negative for CD10 (Fig. 2C), CD117, Dog-1, S100, and CD34, while the endometrial glands and stroma were positive for ER (Fig. 2A) and PR (Fig. 2B) and negative for CD117, Dog-1, S100, CD34, Desmin (Fig. 2D), and SMA (Fig. 2E). The endometrial interstitium was positive for CD10 (Fig. 2C). The expression of Ki-67 in the smooth muscle cell and endometrial glands was very low, with only a few positive cells (<1%; Fig. 2F). According to the immunohistochemical staining results, the final diagnosis of the extrauterine adenomyoma of the lesser omentum was confirmed.

### 2.3. Literature search

We searched the PubMed database with the following search terms: “extrauterine adenomyoma” or “uterus-like mass” or “extrapelvic adenomyoma” or “pelvic adenomyoma”. The paper was limited to articles published in the English language. The following information was manually recorded: patient age, size and location of the extrauterine adenomyoma, history of uterine surgery, symptoms, malignant transformation, and other concomitant tumors. Totally 143 articles were found (Table 1). Initially, through screening the titles and abstracts of these articles, 93 articles were excluded if they were non-English articles, reviews, and not related to extrauterine adenomyoma. Then, 5 articles were further excluded by reading the full text to find if the tumor was not outside the uterus or not related to extrauterine adenomyoma. Finally, 45 articles (53 cases) of extrauterine adenomyoma met the conditions (Table 2).

**Table 1:**
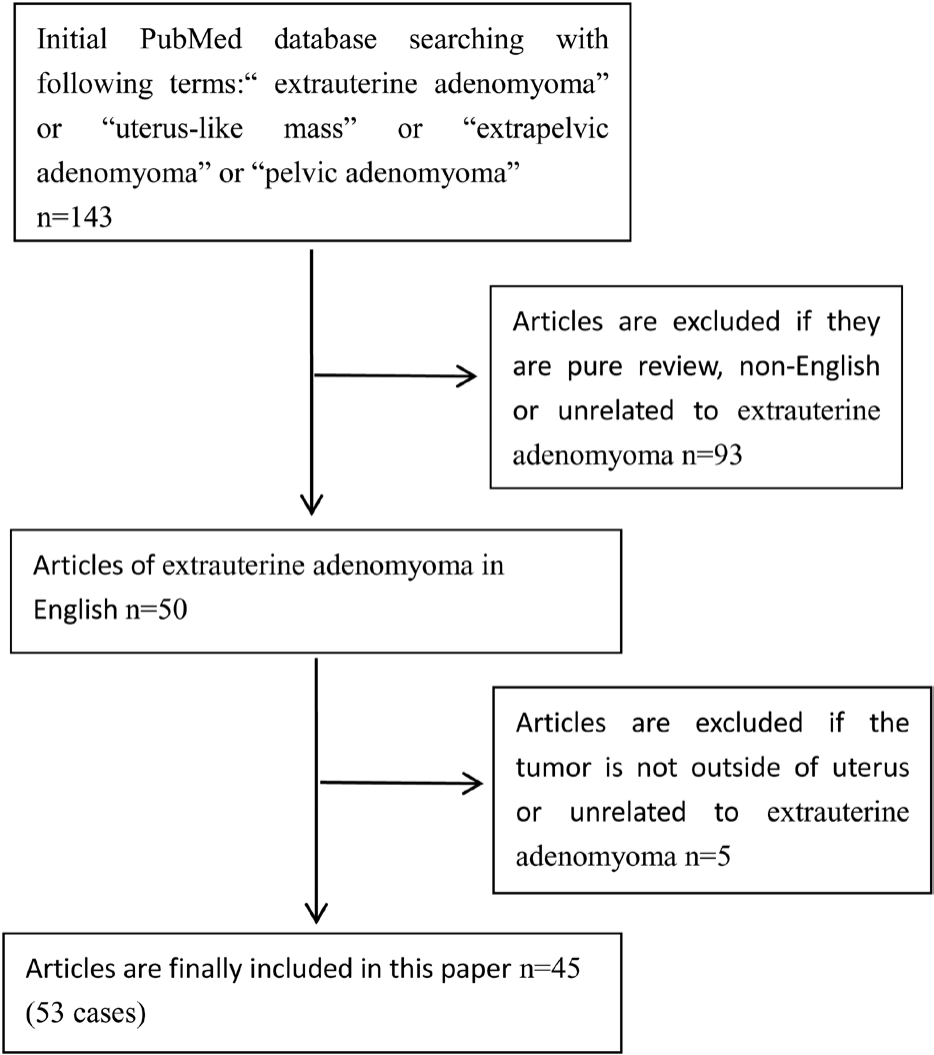
Flowchart for literature search.

**Table 2:** Description of extrauterine adenomyoma.

Sr. no.	Study (year)	Case num.	Age	Size and location	History of uterine surgery	Symptoms	Malignant transformation	Accompanied by other tumors
1	Rubenstein and Kurzon (1952)^[2]^	1	40	5.2 × 3 × 2.5 cm, right round ligament	No	Right groin pain	No	No
2	Rahilly and al-Nafussi (1991)^[7]^	1	38	5 cm, right ovary	No	Right iliac fossa and pelvis pain	Endometrioid adenocarcinoma	No
3	Ahmed et al (1997)^[16]^	1	46	16 × 14 × 11.5 cm, right broad ligament	No	Abdominal pain and bloating	No	No
4	Mitra et al (1997)^[8]^	1	34	2 cm, left ovary	No	Foul smelling discharge	No	No
5	Horie and Kato (2000)^[36]^	1	59	14 × 11 cm, small bowel mesentery	No	Mass lower abdomen	No	No
6	Redman et al (2005)^[3]^	1	50	5 cm, pararectal	Yes	Dysuria and suprapubic, pelvic pain	No	No
7	Bayar et al (2006)^[9]^	1	38	7.5 cm, left ovary	No	Infertility and pelvic pain	No	No
8	Donnez et al (2006)^[4]^	1	48	4 × 4 × 3 cm, left pararectal fossa	Yes	Pelvic pain and dyspareunia	No	No
9	Choudhrie et al (2007)^[25]^	1	57	0.8 cm, left ovarian ligament	No	Lump lower abdomen and pelvic pain	No	No
10	Menn et al (2007)^[17]^	1	37	6 × 4 cm, right broad ligament	No	Right quadrant pain and intermenstrual spotting	No	No
11	Kaufman and Lam (2008)^[28]^	2	39	7 × 5 cm, right pelvic wall	No	Dysmenorrhea, pain, menorrhagia	No	No
			57	10.5 × 9 cm, right pelvic wall	Yes	Right iliac fossa pain, suprapubic pain and backache	No	No
12	Stewart et al (2008)^[10]^	2	40	6 × 4.5 cm, left para- ovary	Yes	Left iliac fossa pain	No	No
			65	6.3 × 4 cm, right parametrial	No	Pelvic mass	No	No
13	Carinelli et al (2009)^[11]^	2	46	10 cm sigmoid, 6 cm pelvic, 4 cm ileal, 1 cm paraileal and paravesical	No	Abdominal pain and constipation	No	No
			39	3 cm sigmoid, 3.5 cm right ovary	No	Dysmenorrhea, Chronic abdominopelvic pain	No	No
14	Api et al (2009)^[12]^	1	45	12 × 6 mm left ovary	No	hypermenorrhea	No	No
15	Mandal et al (2009)^[13]^	1	60	9 × 8 × 8 cm left ovary	No	pelvic pain, tenderness	No	No
16	Liang et al (2010)^[18]^	1	17	4 cm, left broad ligament	Yes	Dysmenorrhea and pelvic pain	No	No
17	Moon et al (2011)^[5]^	1	41	7 × 6 cm, pararectal	No	Asymptomatic	No	No
18	Seki et al (2011)^[32]^	1	44	3.8 × 2 cm, left inguinal region	No	Left inguinal surgical scar periodic pain	No	No
19	Shin et al (2011)^[33]^	1	31	3 × 2.5 × 2.3 cm, adjacent to the sigmoid colon	No	Low abdominal pain	No	No
20	Sisodia et al (2011)^[26]^	1	56	5.5 × 5.3 cm, right ovarian ligament	No	Dysuria, lower abdominal pain, bleeding per vaginum	No	No
21	Khurana et al (2011)^[42]^	1	47	13 × 9 × 5 cm, abdominopelvic region	Yes	Bleeding per vaginum	No	No
22	Carvalho et al (2012)^[39]^	2	32	Few mm to 50 mm, pelvic and abdominal peritoneum and omentum, left ovary	Yes	Asymptomatic	No	No
			41	Few mm to 20 mm, pelvic and abdominal peritoneum and omentum	No	Dysmenorrhea and pelvic pain, proctalgia	No	No
23	Etoh et al (2012)^[27]^	1	45	8 cm, left fallopian tube	Yes	Left low abdominal pain without fever over 3 days	No	No
24	Kim et al (2012)^[35]^	1	46	2 × 1.5 cm, appendix	Yes	Right lower quadrant pain	No	No
25	Moghadamfalahi and Metzinger (2012)^[6]^	1	39	6 cm pararectal, 7.5 cm upper abdomen	Yes	Abdominal pain and bleeding per rectum	No	No
26	Bulut and Sipahi (2013)^[23]^	1	56	5 cm to 10 cm, bilateral broad ligament with pus, ectopic adrenal tissue	No	Menorrhagia and pelvic pain	No	No
27	Wu et al (2013)^[43]^	1	29	3.6 × 2.6 cm, liver	Yes	Back pain	No	No
28	Na et al (2013)^[34]^	1	39	Cecum, descending colon and mesocolon	Yes	Right lower quadrant pain	No	No
29	Nechi et al (2013)^[19]^	1	54	9.3 × 6.3 cm, broad ligament	No	Pelvic pain	No	No
30	Nakakita et al (2014)^[38]^	1	67	6 cm, retroperitoneal space	No	Low back pain	Clear cell carcinoma	No
31	Ulm et al (2014)^[24]^	1	49	3 cm, left round ligament	No	Metromenorrhagia	Endometrioid adenocarcinoma	No
32	Ko and Cheung (2015)^[44]^	1	64	4 cm, right adnexa	No	Asymptomatic	No	Right thigh sarcoma
33	Sopha et al (2015)^[29]^	1	47	1.4 cm, liver	Yes	Right quadrant and back pain	No	Teratoma
34	Torres et al (2015)^[20]^	1	58	4 cm, right broad ligament	No	Postmenopausal bleeding	Clear cell carcinoma	No
35	He et al (2016)^[21]^	1	43	7 × 4.6 cm, left broad ligament	No	Acute lower abdominal pain and hypomenorrhea	No	No
36	Muzykiewicz et al (2017)^[45]^	1	32	15.5 × 14 × 9 cm, extraperitoneal space of the pelvis	No	Asymptomatic	No	No
37	Na et al (2017)^[14]^	3	43	4.7 × 3.3 cm, left adnexa	Yes	Low abdominal pain, dyspareunia, menometrorrhagia	No	No
			36	1.7 cm, left adnexa	No	Low abdominal pain, menorrhagia, hypermenorrhea	No	No
			45	0.8 cm, right ovary	No	Asymptomatic	No	No
38	Tandon et al (2017)^[30]^	1	50	6 × 4.5 cm, liver	Yes	Lower abdominal pain	No	No
39	Paul et al (2018)^[1]^	3	39	10 cm, pararectal	No	Heavy menstrual bleeding, mid cycle pain, and difficulty in initiating micturition	No	No
			45	3 cm, right round ligament	Yes	Right lower quadrant pain	No	No
			37	6 cm pararectal mass, 3 cm ovarian mass	Yes	Subfertility, intermenstrual spotting, dysmenorrhea, constipation	No	No
40	Belmarez et al (2019)^[40]^	1	50	6 mm to 14.5 cm, left ovary, right round ligament, vaginal cuff, bladder, small bowel, and rectosigmoid colon	Yes	Abdominal bloating and indigestion	No	No
41	Liu et al (2019)^[22]^	1	51	7.4 × 4.4 × 3.8cm, right broad ligament	No	Irregular vaginal bleeding	No	No
42	Gruttadauria et al (2020)^[41]^	1	47	3 cm to 7 cm, bilateral uterosacral areas and sigmoid mesentery	Yes	Hip pain	No	No
43	Hsieh et al (2020)^[37]^	1	40	4.5 cm, small intestine	No	Epigastric pain and fullness with intermittent fever	No	No
44	Liberale et al (2020)^[15]^	1	40	6.3 × 6.2 × 6 cm, left ovary	No	Pelvic pain	No	Contralateral serous borderline tumor
45	Ramphal et al (2020)^[31]^	1	44	2.2 × 1.4 cm, right inguinal region	No	Right inguinal region pain	No	No
46	Chen et al (2021) ^Our case^	1	55	0.7 × 0.5 × 0.3 cm, lesser omentum	Yes	Abdominal pain, nausea and vomiting	No	Gastric carcinoma

## 3. Discussion

Adenomyoma is benign tumor composed of smooth muscle cells, endometrial glands, and endometrial stroma.^[1]^ The tumor is most common in the uterine muscle wall, and extrauterine adenomyoma is rare.^[1]^ Rubenstein and Kurzon^[2]^ first reported a case of extrauterine adenomyoma of the right round ligament in 1952. To the best of our knowledge, there are a total of 45 reports (53 cases) in English.^[1–45]^ The most common location for a single mass was in the pelvic cavity (37 cases), including 12 cases in adnexa^[7–15,27,44]^ and 7 cases in broad ligament.^[16–22]^ There were 9 cases of single mass outside the pelvis,^[29–33,35,36,38,43]^ of which 3 cases occurred in the liver.^[29,30,43]^ There are 10 cases of multiple foci.^[1,11,23,34,39–41]^ Here, we introduced the first case of a single extrauterine adenomyoma that occurred in the lesser omentum and further added to the knowledge of this rare finding in a patient with single mass of extrauterine adenomyoma outside the pelvis.

The age of patients, including our report case, was 17 to 67 years old, and most showed different clinical symptoms (48/53). Nineteen of 53 patients had a history of uterine surgery.^[1,3,4,6,10,14,18,27–30,34,35,39–43]^ The causes of surgery included uterine leiomyoma, adenomyosis, irregular vaginal bleeding, cesarean section, and cervical lesions. This patient had undergone a hysterectomy for uterine leiomyoma 8 years ago.

The general gross characteristics of extrauterine adenomyoma are nodules with clear boundaries.^[1]^ However, it is difficult to distinguish extrauterine adenomyoma from leiomyoma, gastrointestinal stromal tumors, and similar conditions by using imaging examination in the clinic.^[1]^ In our case, the lymph node-like mass in the lesser omentum failed to attract the special attention of the imaging doctor and was disregarded as lymph nodes. Even from resected lesser omentum, initially, the mass was disregarded as lymph node with cancer metastasis. The final diagnosis was obtained by histological examination according to the typical histological features. This lymph node-like mass was shown to be composed of smooth muscle tissue, benign endometrial glands, and stroma. Therefore, the diagnosis of extrauterine adenomyoma was initially established. However, the extrauterine adenomyoma needs to be differentiated from leiomyoma, gastrointestinal stromal tumor, schwannoma, granulosa cell tumor, fibroma, and similar conditions.^[1,26]^ The smooth muscle of adenomyoma has characteristic positive expression of ER,^[43]^ PR,^[43]^ SMA,^[43]^ and Desmin^[43]^ and negative expression of CD117, Dog-1, CD34, and S100. Leiomyomas that originate in the gastrointestinal tract or omentum positively express SMA and Desmin and negatively express ER, PR, CD117, Dog-1, CD34, and S100, and the tumors are all composed of smooth muscle cells without endometrial components. Gastrointestinal stromal tumors are mostly positive for CD117, Dog-1, and CD34. Schwannomas and granulosa cell tumors are mostly positive for S100. The endometrial glands of extrauterine adenomyoma show characteristic positive expression of ER and PR, while the endometrial stroma of extrauterine adenomyoma is characteristically positive for CD10, ER, and PR.^[43]^ Our case is fully consistent with the typical performance, and therefore, the final diagnosis of the extrauterine adenomyoma was confirmed.

Although the pathogenesis behind extrauterine adenomyomas is not well understood, several proposed theories have been published.^[1,3,6,10–13,15,16,18,23,25,26,28,29,33,40–43,46–50]^ “Smooth muscle cell metaplasia” theory, it was first proposed by Cozzutto that adenomyomas form when there is a focus of endometriosis which undergoes metaplasia into smooth muscle.^[47]^ Our case was without a history or evidence of endometriosis. “Seeding” theory, it is stated that the tumor could arise from deposits of iatrogenic dropped cells within the abdomen and pelvis.^[40]^ However, not all patients with extrauterine leiomyomas or adenomyomas have a history of their intrauterine counterparts. In our patient, there was a history of uterine leiomyoma, but none of adenomyosis or adenoma inside or outside of uterus was found. “Defective Müllerian duct fusion” theory, it was proposed by Rosai et al^[50]^ that abnormalities of the uterus, such as rudimentary horn or uterine duplications could lead by a process of detachment to an implant of a uterus-like mass in the abdominal cavity.^[1,3,12]^ Our case did not have accompanying urinary abnormalities like renal agenesis, therefore the pathogenesis of this case could not be explained by this theory. “Subcoelomic mesenchymal metaplasia” theory, it was proposed by Redman et al^[3]^ that multipotent cells, contained below the mesothelial layer of the peritoneum, could differentiate and grow under hormonal (estrogen) prompting, leading to the formation of a supernumerary Müllerian uterus-like structure.^[11,18]^ Our case did not accept the hormonal therapy and it seems not belong to this theory. “Mullerianosis” theory, it was proposed by Batt^[46]^ that the heterotopic Müllerian-like organoid tissue of embryonic origin could develop within other normal organs during organogenesis. This theory could be particularly suitable for providing an explanation for extrauterine lesions that occurred in unusual sites outside the pelvic and lower abdominal cavities. It seems that our patient could be somehow explained by this theory. Nevertheless, further work will need to be done and more cases will need to be reviewed to explore which theories are correct. Finally, it should point out for our case that it seems no causal relationship existing between extrauterine adenomyoma of the lesser omentum and stomach adenocarcinoma.

Previous reports have shown that extrauterine adenomyoma could also undergo malignant transformation, such as to endometrioid adenocarcinoma^[7,24]^ or to clear cell carcinoma.^[20,38]^ So far, there has been no report of the malignant transformation of extrauterine adenomyoma into endometrial stromal sarcoma and leiomyosarcoma, but it is possible in theory. Therefore, if the cells of extrauterine adenomyoma appear significantly atypia, we must be alert to the possibility of malignant transformation. The morphology of smooth muscle, glands, and endometrial stroma, in this case, was no any sign of atypia, and Ki-67 immunohistochemistry showed very little proliferation activity; therefore, this was a benign extrauterine adenomyoma. Furthermore, especially as our case, we should pay attention to avoiding misdiagnosis of this rare extrauterine adenomyoma of the lesser omentum as lymph node adenocarcinoma metastasis.

## 4. Conclusion

In summary, the extrauterine adenomyomas that occurs outside the pelvic cavity are extremely rare. We present the first case of a single extrauterine adenomyoma of the lesser omentum. Such mass could be easily misinterpreted in the preoperative imaging examination and even in the gross examination of resected specimens postoperatively. Pathological examination is ultimately required to confirm the diagnosis. The pathogenesis of extrauterine adenomyoma of the lesser omentum in this report is not quite clear; it may relate to Mullerianosis. Since some of these tumors may undergo malignant transformation, when the cells in the tumor appear atypical, we must be alert to the possibility of malignancy. A possible misdiagnosis of this rare extrauterine adenomyoma of the lesser omentum as lymph node adenocarcinoma metastasis should also need to be avoided.

## Author contributions

Conceived and designed the project: Yanlin Chen, Zhong Zuo and Tianwen Luo

Worked up the case: Yanlin Chen

Literature searching: Yanlin Chen and Liangyong Deng

Writing—original draft: Yanlin Chen and Liangyong Deng

Writing—review and editing: Jingbo Zhao

Approved the final manuscript: All authors

## Acknowledgments

We are thankful for grants from the Chongqing Science and Health Joint Medical Research Project (No. 2018ZDXM010), the Cultivation Fund of the First Affiliated Hospital of Chongqing Medical University (No. PYJJ2018-28), and the Cultivation Fund of the Women and Children’s Hospital of Chongqing Medical University (No. 2021YJMS01).
